# 

**Published:** 1857-12

**Authors:** 


### OUR EXCHANGES.

We ask of our exchanges the courtesy of inserting for us the list of contents of our December number, our terms, and our address,—if they think our *Journal* merits, in the main, a liberal subscription list. One thing we wish to boast of, we never use our influence against our holy religion, but always on its side; this, however, is not the fashion of the times among publications, not professedly religious. Too many have gained an influence, and then give a traitorous thrust whenever they can make by it—not always making, however, we are glad to know. "Putnam" tried that game, and perished soon after. One of the most popular British Quarterlies tried it, and had to turn, to save its life. We think the religious press, with a few exceptions, have committed grave errors in their wholesale commendations of books and periodicals; and we suggest that, hereafter, only the articles critically read be spoken of, or that simply a list of the contents be given, without note or comment. For ourselves, we pay no sort of attention to "reviews and notices," they nearly always mislead us; but we have a thousand times bought a book, or pamphlet, or paper, for a single item in its list of contents. This is a practical suggestion, worthy the attention of all publishers. We had rather see a bald list of the contents of one of our numbers in a newspaper, than a whole column of commendations. *Will our exchanges please note this?* We will serve them in the same way. And that there is weight in this, we notice a suggestive fact. In several of our exchanges, there have appeared friendly notices of our Journal, as regular as the month came round, *for years!* without its bringing one single subscriber from that locality!! Does not this show what little weight is attached to an editorial puff? and is it not humiliating? Within the last six months, our subscription list has *doubled* ITSELF. We have had no paid agents, and have had no leisure, nor taken any pains of a special character to extend our circulation, hence, we truly say, our list lacks but fifty of *doubling itself.* We have double the number of subscribers we have ever had before, and have no delinquents. Nearly every new subscriber is a volunteer. The letter runs thus, and is almost stereotyped: "Having seen frequent extracts from '*Hall's Journal of Health,*' and they being always good, short, and to the point; and having just learned where it is printed, I herewith inclose you the subscription for one year."

*Brother Editors!* Attention! If you want to "hub us," if you want to help us along, don't fool away your time and waste your brains in saying complimentary things, for it don't pay either of us; simply give our list of contents, and we will do as much for you now and then.

### OUR RELIGIOUS EXCHANGES.

Few people imagine how much useful, solid reading there is in a single Religious Newspaper: no such paper ought to be destroyed, unless it be for the purpose of cutting out and preserving some of the most important pieces. We have many times copied an article, rather than mutilate the paper. But we do not lay the papers carefully away, to be eaten with the mould of years—that is hiding a talent in the earth; it is locking money up in a drawer, benefiting nobody, while its use is lost to the world, its interest is wasted. We carefully lay our religious exchanges away, and have a worthy man, Mr. Joseph Harris, to come at the first of the month, and distribute them among the sailors of ships which are just leaving port; and in one instance, among many, when a vessel returned from a long voyage, the sailors deputized one of their number to wait on Mr. Harris, to express their heartfelt obligations for the enjoyment they had derived from the reading matter which he had furnished them, and then presented him with a handsome sum of money as an inducement to him to continue in his work of benevolence, in distributing religious papers, tracts, and books, thus.

And in this connection, the thought has often occurred to us, what comfort, what encouragement, what cheer has a newspaper article, of even half a dozen lines, written with spirit, vigor, and point, carried to the heart of the lonely sailor

"Far at sea!"

or to the prisoner in his dreary dungeon! for some of our papers are sent there also. We tell you, reader, that man has lived to purpose, who has penned for a paper three lines of stirring thought. Thus it ought to be considered a privilege to be allowed to write for a religious newspaper, provided there is the talent to do it well.

Let the clergy, then, and all persons of intellect, leisure, and a heart for good, make it a *weekly* task to compose a few lines for their favorite paper—lines which paint some burning thought as it leaps from the brain, keen as a Damascus blade; and which wells up from the heart, all luscious with the love of human kind—a thought which shall kindle up humanities in the living now, scattered over land and sea, and will continue to do it, may be, until the last wave of time has been lost in Eternity's ocean.

We had rather be the writer of an eight or ten lined paragraph, thought worthy of being quoted in the *American Messenger*, or the *Illustrated Christian Almanac*, with their half million readers, than to be the author of any volume ever published by the "Great Unknown," or the immortal "Boz"—a paragraph for enduring good, not for the glittering glory of an hour, and as false as it is fair.

In doing good we should study economies; for nothing should be wasted in this great world of want. To make a paper last long, and thus serve a good many readers, it should have as few folds as possible. An eight-paged newspaper is torn up or worn out in a very short time; besides, there is too much reading in it, it is not remembered, and many excellent items are skipped over. So we make of those two excellent papers, the *Presbyterian*, and the *American Presbyterian* of Philadelphia, with their large, attractive headings of short articles, what is equal to four tracts; and of the *New York Evangelist*, with its eight pages of useful truth, weekly repeated, with its name and date heading each page, no less than four tracts, to be read by four different persons at a time. We treat *The New York Observer* in the same way, although it has never been willing to exchange with us; yet, as it has given us a good many subscribers by quoting from our Journal, for weeks in succession, we can afford to pay for it—the proprietors being *poor!* For all that, *The New York Observer*, by its steady and powerful conservatism, in matters of State as well as Church, has worked a good for our church and nation beyond that of any periodical in America: and long may it and the *National Intelligencer*, and papers of their sort, remain the Malakoffs of our religion and our nation!

As to our *Agricultural* exchanges, they are never wasted, but are given to our patients, or sent, by mail, to our friends and acquaintances, whom we think might be benefited by subscribing for them. It is gratifying for us to state the fact, that every Agricultural Exchange on our list is always on the side of morals and religion. We never recollect to have seen an article in one of them unfit to be seen at the family fireside.

Of our exchanges in pamphlet form, one of the best is *Littell's Living Age*, of Boston, an 8vo weekly, at six dollars a year, being made up of the best foreign articles of a philosophical, literary, and miscellaneous character. It makes a large standard volume every year. It is sustained by the Aristocracy of Letters. One little item may be here mentioned: articles from our Journal find their way to England, and are then quoted in Littell, as coming from European publications. Does it require that an article to be good and new must cross the Atlantic twice? But we are placated in the reflection that what "*Dr. Hall in his Journal says*," is appreciated abroad as well as at home, as being useful and true.

As to our Monthly Exchanges, there is *The Mother's Journal* for parents; *Merry's Museum* for children; *Arthur's Home Magazine*, ALWAYS *safe* reading for the family; *Godey's Lady's Book*, that in a quarter of a century has never insulted any man's religion: all these are useful in their way, and merit public consideration. *Graham* has a nation of friends.

### SPECIAL NOTICE.

THIS number closes the year for which all our subscribers have paid. Our terms are, "IN ADVANCE." We do not know how many on our present list will wish to continue; and as we do not want to publish a greater number of copies, for January, than will be taken—and as the next number must be sent to the press by the 15th of December—we hope that such of our friends as desire to continue with us another year, will send us their name and dollar, immediately on the receipt of this number. It is a little thing—can be attended to in any five minutes—and then *it is off your mind at once*, and for a whole year.

The safest and best way to remit is : say nothing about it to any body, but take a gold dollar, fold it over at one corner of your letter, and sew around it ; put it in an envelope, seal it with a wafer, put on a postage stamp, and DEPOSIT IT IN THE POST-

OFFICE YOURSELF: we have never known a money-letter miscarry in all our publishing experience—nor have we ever known a medical fee to miscarry in a practice of many years—when thus attended to.

We do not hesitate to say, that nine out of ten of all the losses of money, by mail, complained of by hasty and inconsiderate editors, are owing to the dereliction of the person who sends the money, or the person to whom it is sent. If this is not so, how is it that we never lose any money by mail, not a dozen dollars in the receipt of thousands, in small sums, scattered over many years? We attribute it to *one fact mainly:* we have always made it a practice *to take our letters out of the office ourselves.* We have done it in New York; we did it in Cincinnati and in New Orleans; and the result was the same at each place: in fifteen years, we do not remember to have lost as many dollars—certainly, we have not lost one dollar in a thousand.

If a subscriber cannot get a gold dollar, then send a current note of this city or State; if you cannot get such a one, then send a note current with you, with as many postage stamps as will make it a dollar at New York; for our terms are, a full dollar a year, in advance.

We would like every clergyman to have the reading of our Journal; but many of them are too scantily paid to afford it. How many of our subscribers feel interested enough in the gratification and health of a minister of their acquaintance to send an extra dollar for such a one.

During 1857, we received more extra dollars for this purpose from the South than from any other direction. What is the reason?

We will send, post paid:

| | |
|---|---|
| "Bronchitis and Kindred Diseases," for | $1 00 |
| "Consumption," for | 1 00 |
| Bound Volumes of Journal, I., II., III., IV., | 5 00 |
| Journal for 1858, not post-paid, | 1 00 |

The prices are the same if purchased at this office.

☞ All subscriptions and money for books must be sent to—address simply—

"DR. W. W. HALL,
"New-York."

